# Postbiotic-Based Extracts from Native Probiotic Strains: A Promising Strategy for Food Preservation and Antimicrobial Defense

**DOI:** 10.3390/antibiotics14030318

**Published:** 2025-03-18

**Authors:** Diana Molina, Ioana C. Marinas, Evelyn Angamarca, Anamaria Hanganu, Miruna Stan, Mariana C. Chifiriuc, Gabriela N. Tenea

**Affiliations:** 1 Biofood and Nutraceutics Research and Development Group, Faculty of Engineering in Agricultural and Environmental Sciences, Universidad Técnica del Norte, Av. 17 de Julio s-21 y José María Córdova, Ibarra 100150, Ecuador; 2 Research Institute of the University of Bucharest—ICUB, University of Bucharest, 050095 Bucharest, Romania; ioana.cristina.marinas@gmail.com (I.C.M.); miruna.stan@bio.unibuc.ro (M.S.); carmen.chifiriuc@unibuc.ro (M.C.C.); 3 Department of Inorganic and Organic Chemistry, Biochemistry and Catalysis, Faculty of Chemistry, University of Bucharest, 050663 Bucharest, Romania; 4 “C. D. Nenitzescu” Institute of Organic, Supramolecular Chemistry of the Romanian Academy, 060023 Bucharest, Romania

**Keywords:** postbiotics, lactic acid bacteria, antioxidant capacity, cytotoxicity, antimicrobial activity

## Abstract

**Background/Objectives**: The deterioration of food quality and safety is often linked to the presence of pathogenic and spoilage microorganisms. Postbiotics, including organic acids, enzymes, and bacteriocins produced by lactic acid bacteria (LAB), have emerged as promising next-generation food preservatives. This study investigates the biological and physicochemical properties of several postbiotic-based extracts (PBEs) comprising cell-free supernatant (CFS) and exopolysaccharide (EPS) fractions derived from three native probiotic strains: *Lactiplantibacillus plantarum* UTNGt2, *Lactococcus lactis* UTNGt28, and *Weissella cibaria* UTNGt21O. **Methods:** The antibacterial activity of these PBEs was assessed against multidrug-resistant *Escherichia coli* L1PEag1. Moreover, the antioxidant capacity and cytotoxicity along with the characterization of these formulations was assessed. **Results:** FU6 (CFS UTNGt28: EPS UTNGt2) and FU13 (CFS UTNGt21O) were found as the most potent formulations. Transmission electron microscopy (TEM) and scanning electron microscopy (SEM) confirmed dose- and time-dependent damage to the bacterial membrane and cell wall. FU6 exhibited superior antioxidant activity and lacked hemolytic effects, whereas both FU6 and FU13 induced cell-specific responses in HEK293 (human kidney) and HT-29 (intestinal mucus-producing) cell lines. Furthermore, attenuated total reflectance-Fourier transform infrared (ATR-FTIR) spectroscopy identified characteristic absorption bands corresponding to proteins, lipids, carbohydrates, and nucleic acids, while proton nuclear magnetic resonance (^1^H-NMR) spectroscopy revealed key monosaccharides, amino acids, and metabolites such as lactate and acetate within the extracts. **Conclusions:** FU6 and FU13 demonstrate potential as safe and effective postbiotic formulations at non-concentrated doses. However, further research is required to elucidate their molecular composition comprehensively and evaluate their applicability for broader and long-term use in food preservation and pharmaceutical development.

## 1. Introduction

The decline in quality and reduced storage time of foods are often linked to the presence of pathogenic and spoilage microorganisms [1]. The accumulation of harmful substances and their adverse effects on human health have highlighted the urgent need to develop new antimicrobial bioformulations to ensure food safety throughout the entire supply chain, from raw material processing to transport, distribution, and storage [2].

In recent years, microbial metabolites derived from lactic acid bacteria (LAB) have garnered increasing attention due to their substantial applications in the food industry [1,3]. Probiotic LAB produce a variety of bioactive compounds during fermentation which exhibit a range of beneficial properties such as pathogen inhibition, immune system modulation, and anti-inflammatory effects [4]. These compounds are denominated postbiotics, an emerging class of biotics with the potential to provide health benefits [5]. Postbiotics are defined as a “preparation of inanimate microorganisms and/or their components that confer a health benefit on the host” [6]. According to the International Scientific Association of Probiotics and Prebiotics, postbiotics include (i) soluble factors such as enzymes, peptides, teichoic acids, peptidoglycan-derived muropeptides, polysaccharides, cell-surface proteins, and organic acids, which are secreted by live bacteria or released upon bacterial lysis; (ii) non-viable microbial metabolites that exert biological effects on the host; and (iii) compounds produced by microorganisms, derived from food components or microbial constituents, including non-viable cells, that promote health and well-being when administered in appropriate amounts [6]. Unlike probiotics, which depend on the activity of live microorganisms, postbiotics can be added to foods as supplements, providing health benefits through their bioactive metabolites without requiring viable bacteria. Additionally, postbiotics exhibit antioxidant and antimicrobial properties that can enhance food safety and extend shelf life of food products [5]. However, the antimicrobial effects of postbiotics have been primarily documented in vitro against pathogens from international collections [7,8], with limited research assessing their efficacy against multidrug resistant pathogens originating from food and testing their performance ex vitro through direct application to food materials.

Beyond antimicrobial activity, postbiotics have shown potential for boosting innate immunity, treating gut disorders, and supporting overall gut health [9,10]. Studies have also demonstrated the ability of various LAB-derived compounds, such as exopolysaccharides, to exhibit antitumor effects against colon carcinoma cells [11]. As a result, postbiotics are emerging as a promising alternative for maintaining the microbiological safety of raw and minimally processed foods post-harvest, either independently or in combination with edible coatings [12,13]. Notably, the active compounds in postbiotics are safe for human health [14].

Recent research has highlighted the potential of native LAB strains isolated from Ecuadorian Amazon fruits to produce postbiotics [15,16]. Furthermore, cell-free supernatants (CFS), peptide-proteins (PPs), and exopolysaccharide (EPS) extracts from these LAB strains have been shown to inhibit pathogens [16]. Enhanced antimicrobial activity was observed when combining CFS from different LAB species in different ratios [17]. Understanding the antimicrobial mechanisms against multidrug resistant pathogens and evaluating the cytotoxicity and antioxidant capacity of these compounds are crucial steps toward developing postbiotic-based antimicrobial products for use as novel preservatives or food supplements.

The aim of this study was to select the most efficient antimicrobial postbiotic-based extracts (PBEs) derived from three native bacteriocinogenic and probiotic lactic acid bacteria (LAB) strains and characterize their bioactive properties. The cytotoxicity of two selected extracts, FU6 and FU13, was assessed in human embryonic kidney cells (HEK293) and goblet-like mucus-producing intestinal cells (HT-29-MTX). Their antibacterial activity was evaluated against a multidrug-resistant *E. coli* L1PEag1 strain, alongside an analysis of their antioxidant properties to support their multifunctional potential for food preservation. Additionally, attenuated total reflectance-Fourier transform infrared (ATR-FTIR) spectroscopy and proton nuclear magnetic resonance (^1^H-NMR) spectroscopy were employed to determine their chemical composition. Finally, the potential mechanisms of antimicrobial activity were explored by assessing their effects on bacterial membrane integrity and ultrastructural morphology.

## 2. Results and Discussion

### 2.1. PBEs Co-Cultivated with L1PEag1 Inhibit the Cell Viability in a Dose-, Time-, and Producer Strain-Dependent Manner

**Figure 1:**
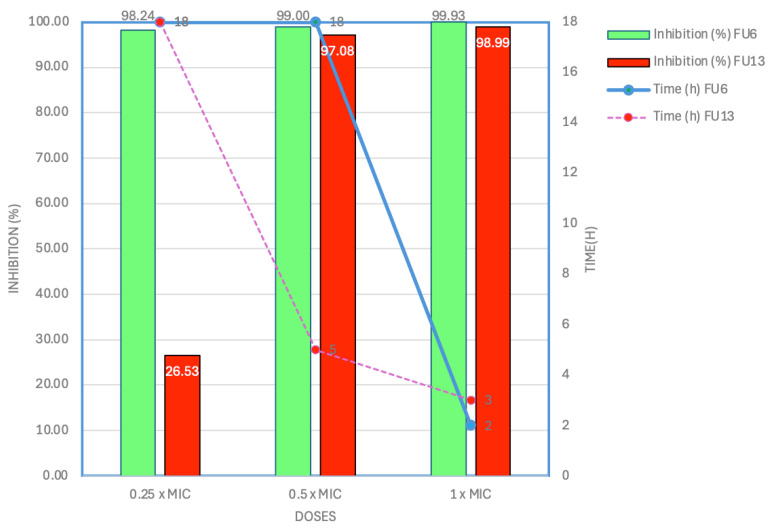
Killing effect of FU6 and FU13 over *E. coli* L1PEag1 at different doses. FU6: CFS of UTNGt28 and EPS of UTNGt2 in proportion 3:1 (*v*/*v*); FU13: CFS of UTNGt21O. CFS: cell-free supernatant; EPS: exopolysaccharides.

Antibiotic-resistant, human pathogenic *E. coli* strain L1PEag1 was isolated from cape gooseberry fruits [18]. Avoiding exposure to antibiotic-resistant microorganisms through ready-to-eat fruits can be challenging due to the interconnected nature of food security and safety concerns. Therefore, it is essential to identify effective fruit protectors that can inhibit the growth, colonization, and spread of these microorganisms during both pre-harvest and post-harvest fruit processing. From the screening antimicrobial results, FU6 and FU13 showed the highest inhibitory activity against L1PEag1 (Table S1). The MIC was 0.25 mg/mL for FU6 and 0.5 mg/mL for FU13 the recorded bacterial growth reduction being >90% at 18 h (Table S2). Time-killing assays indicated that at the 0.25 × MIC, FU6 inhibited the target bacteria by 98.25%, whereas FU13 showed poor inhibitory activity (26.53% cell viability reduction) upon 18 h of incubation (Figure 1). Interestingly, at 0.5 × MIC, FU6 requires 18 h to reduce the cell viability by 99%, whereas FU13 required only 5 h, suggesting that the L1PEag1 cells inhibition is time-, dose-, and composition-dependent. Both FU6 and FU13 showed high inhibitory activity upon 2 h and 3 h, respectively, exposure at 1 × MIC. No effect was observed in the control samples. The slight variation could be attributed to differences in the composition of the ingredients in the formulation, despite the nearly identical inhibition spectra of the two PBEs.

According to early research, the inhibitory activity of compounds that resemble bacteriocin from various species of *L. plantarum* varies by species [19]. In addition, probiotic *W. cibaria* inhibited bacterial growth and biofilm formation by decreasing the production of exopolysaccharides and auto-aggregation, increasing co-aggregation, and downregulating virulence factors [20]. From the whole genome analysis, we showed that UTNGt21O harbored a plethora of genes encoding for several metabolites, acids, and bacteriocins which might contribute to the overall antimicrobial activity against several pathogens [21]. We also showed that UTNGt21O harbored a putative enterolysin_A gene. In addition, the combination of CFS extracts from UTNGt2 and UTNGt28 strains showed high capacity to inhibit both *Staphylococcus aureus* ATCC1026 and *Citrobacter freundii* UTNB3Sm1 [16]. From the EggNOG functional genome analysis, the UTNGt2 strain harbored genes that are associated with capsular polysaccharide production; their function has not been yet investigated. According to early studies, the EPS performs several biological tasks, including facilitating interactions between bacteria and their surroundings, protecting against adverse conditions like dehydration, reducing the effects of toxic stressful conditions, immune response evasion, and phage attack [22,23]. The UTNGt28 strain contains genes encoding sactipeptides and enterolysin A, with the latter also previously identified in the UTNGt21O strain [20]. Notably, at 1 × MIC, rapid bacterial death within 2 h suggests a potential synergistic effect between metabolites in the CFS and EPS, contributing to the observed antimicrobial activity. This hypothesis warrants further investigation. These findings reinforce our earlier conclusion that the antibacterial efficacy varies depending on the strain and the target pathogen.

### 2.2. Interaction of PBEs with E. coli L1PEag1 Led to Alterations in Cell Morphology and Ultrastructure

**Figure 2:**
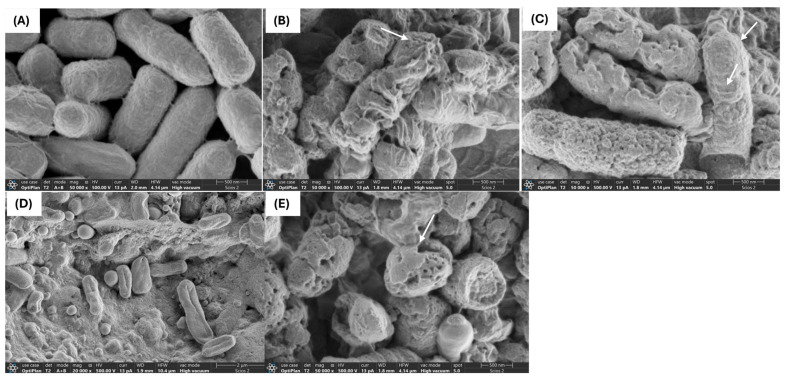
SEM of *E. coli* L1PEag1 treated with PBEs. Legend: (**A**): L1PEag1 untreated cells (exponential phase); (**B**) L1PEag1 treated with 0.5 × MIC FU6; (**C**). L1PEag1 treated with 0.5 × MIC FU13. (**D**) EPS Gt2 only; (**E**) Gt28 only; white arrows indicated cell disruptions which were caused by FU6 and FU13 (disappearance of membranes, broken cells, cell holes. FU6: CFS of UTNGt28 and EPS of UTNGt2 in proportion 3:1 (*v*/*v*); FU13: CFS of UTNGt21O. CFS: cell-free supernatant; EPS: exopolysaccharides; MRS: MRS broth.

**Figure 3:**
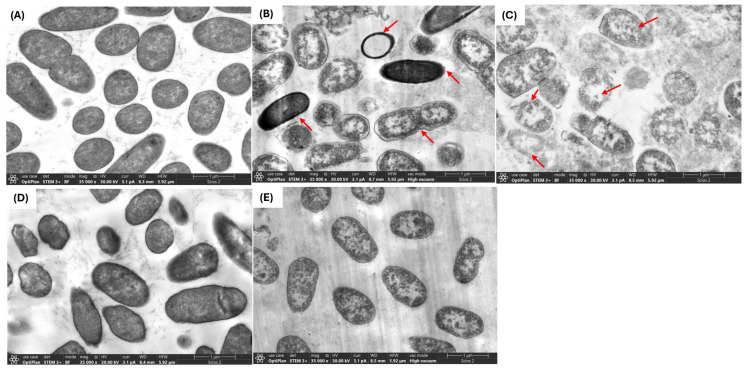
TEM of *E. coli* L1PEag1 treated with PBEs. Legend: (**A**): L1PEag1 untreated cells (exponential phase); (**B**) L1PEag1 treated with 0.5 × MIC FU6; (**C**). L1PEag1 treated with 0.5 × MIC FU13. (**D**) EPS Gt2 only; (**E**) Gt28 only; Red arrows indicated cell disruptions which were caused by FU6 and FU13 (disappearance of membranes, leakage of contents and ghosts, DNA relaxation, separation of cell envelope, spheroplasts). FU6: CFS of UTNGt28 and EPS of UTNGt2 in proportion 3:1 (*v*/*v*); FU13: CFS of UTNGt21O. CFS: cell-free supernatant; EPS: exopolysaccharides; MRS: MRS broth.

The use of antimicrobial compounds produced by LAB to combat the growth of drug-resistant microorganisms in food is a challenging and intricate task. Critical factors such as the type and concentration of the antimicrobial agent, along with the exposure duration, play a pivotal role in influencing the morphological and ultrastructural changes in cells resulting from direct interaction with the compound [24]. Through SEM and TEM analyses, we investigated the effects of FU6 and FU13 on the morphological and ultrastructural changes in *E. coli* L1PEag1 cells. SEM imaging of untreated L1PEag1 cells revealed smooth, intact, rod-shaped structures (Figure 2A). In contrast, cells treated with FU6 and FU13 exhibited noticeable damage, including cell breakage and visible holes (Figure 2B,C). Treatment with EPS Gt2 did not result in significant morphological changes; the EPS biomass merely covered the cells without altering their shape (Figure 2D). However, when Gt28 was applied alone, it induced visible morphological changes in the cells (Figure 2E), suggesting that the effect of FU6 is due to the antimicrobial components released in the CFS, not the EPS. Similarly, TEM analysis showed intact cell walls of untreated cells (Figure 3A), whereas the cells treated with FU6 showed spheroplasts formation, DNA relaxation, separation of the cell’s envelope, and ghost cells (Figure 3B). Likewise, FU13 treatment cells resulted in cytoplasm content released, DNA relaxation, and disappearance of membrane (Figure 3C). No changes were observed upon the treatment of cells with EPS of Gt2, whereas DNA relaxation, separation of the cell’s envelope was shown by CFS Gt28 (Figure 2D,E). These types of events were previously observed when treated *Citrobacter* with several peptide combinations derived from native probiotics [16]. In previously published reports, a chimeric peptide developed to neutralize the growth of multidrug resistant *E coli* showed disrupted cells in TEM analysis [25]. In a similar study, CFS from *L. pentosus* strain L-36 applied at 1 × MIC induces pores formation in the *Staphylococcus aureus* membrane increasing their permeability and subsequently leading to the leakage of nucleic acids and other cytoplasm components [26]. Likewise, Ozma et al. [27] showed that CFS from three probiotic strains lowered the relative expression of virulence genes (*fim*H, *agg*R) of multidrug resistant *E. coli*. This study is the first to demonstrate visible “holes” in the cell membrane of antibiotic-resistant *E. coli* following treatment with an antimicrobial formulation derived from LAB metabolites. The interaction between the formulation and the bacterial cells compromises membrane integrity, resulting in cell rupture, leakage of intracellular contents, and ultimately cell death. This effect is strongly influenced by the formulation’s composition and its specific targets. We propose that metabolite-based extracts derived from native LAB offer a promising approach for developing potent antimicrobial agents. These agents could effectively inhibit the growth and colonization of foodborne multidrug-resistant microorganisms, significantly enhancing food safety and extending product shelf life.

### 2.3. Cytotoxicity Evaluation of PBEs

Assessing potential risks to human health is critical for products intended for use in food, pharmaceuticals, or pesticides [28]. While cytotoxic agents effectively target rapidly dividing cancer cells, they can also damage normal rapidly dividing cells, rendering certain healthy tissues particularly vulnerable to agent-induced cell death [29]. In this study, the observed results are likely due to the distinct biological responses of the two cell lines (HEK293 and HT-29) to FU6 and FU13, as well as the varying sensitivities of the MTT and LDH assays. Specifically, the MTT assay measures mitochondrial activity, whereas the LDH assay assesses membrane integrity. The selected incubation periods were designed to replicate physiologically relevant exposure scenarios for both the colon and kidney. For colonic exposure, the 4 h incubation reflects the initial interaction of PBEs with the mucus layer and colonic epithelial cells during transit through the large intestine, while the 24 h incubation accounts for prolonged exposure. This duration considers the slower colonic transit time (12–48 h) and the potential persistence of PBEs within the mucus layer [30,31]. These timeframes enable the evaluation of immediate cellular responses as well as extended effects on colonic goblet-like cells. For renal exposure, the 4 h incubation simulates the rapid interaction of PBEs with kidney cells, representing the initial filtration and transit through the renal tubules. In contrast, the 24 h period captures prolonged exposure, considering the potential for retention or reabsorption of PBEs in renal tissues under specific physiological or metabolic conditions. These carefully chosen durations provide insight into both short-term and extended cellular effects [32].

**Figure 4:**
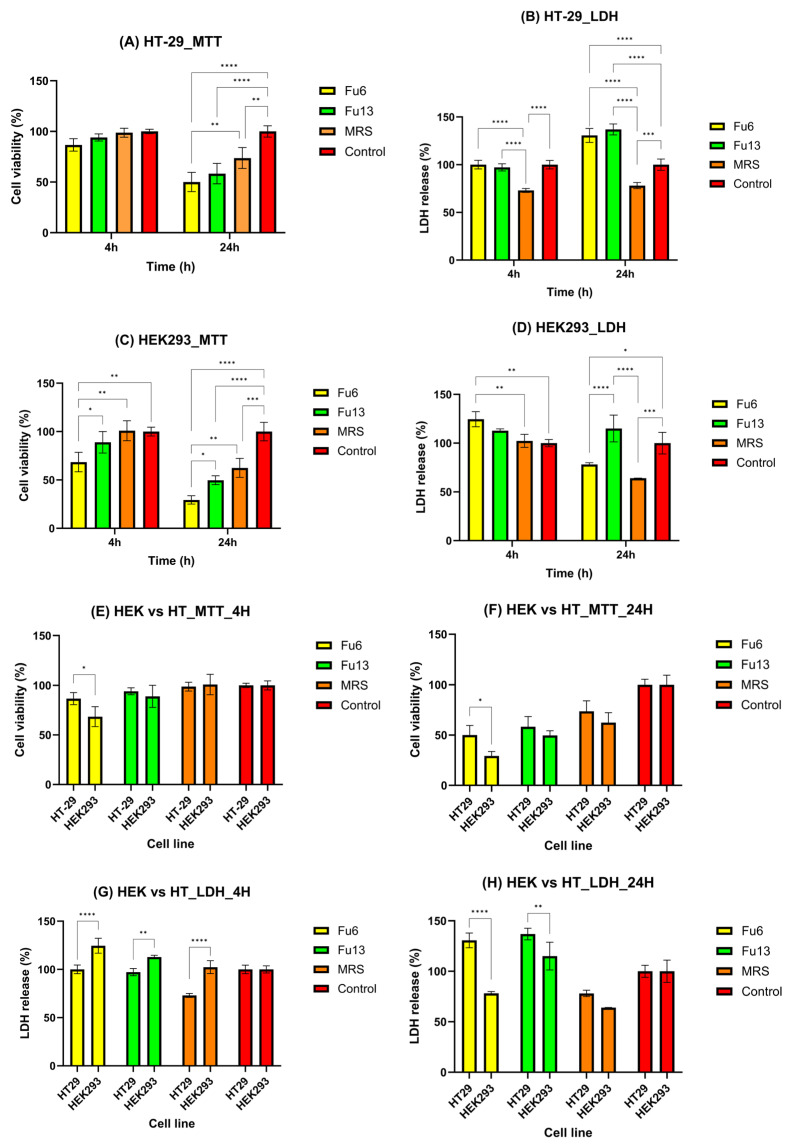
Biocompatibility and cytotoxicity assessment of metabolites extracts using MTT (**A**,**C**,**E**,**F**) and LDH (**B**,**D**,**G**,**H**) assays. Data are presented for HT29 (**A**,**B**) and HEK298 (**C**,**D**) cell lines under standard cultivation conditions, with comparative analysis between the cell lines at different time intervals (**E**–**H**). FU6: CFS of UTNGt28 and EPS of UTNGt2 in proportion 3:1 (*v*/*v*); FU13: CFS of UTNGt21O. CFS: cell-free supernatant; EPS: exopolysaccharides; MRS: MRS broth. Statistical analysis using ordinary two-way ANOVA with Tukey’s multiple comparisons test showed significant differences between samples and culture media, indicated by * *p* < 0.05, ** *p* < 0.01, *** *p* < 0.001, and **** *p* < 0.0001.

The first 4 h of exposure did not induce any important changes in the viability of HT-29 cells, or their membrane integrity compared to the control group, suggesting that FU6 and FU13 did not immediately affect mitochondrial function or metabolism (Figure 4A,B). However, after 24 h, significant cytotoxic effects were detected in both assays, indicating cumulative damage over time, affecting both mitochondrial activity and membrane integrity (Figure 4A,B). In the case of HEK293 cells, a time-dependent decrease in cell viability was noticed (Figure 4C). A slight increase in LDH release after 4 h of incubation with FU6 confirmed an early cell membrane damage (Figure 4D). Furthermore, after 24 h, the LDH values cannot be reliable for membrane damage, as a low percentage of viable HEK-293 cells were detected compared to control. FU6 appeared more cytotoxic to HEK293 cells at 24 h (as indicated by both MTT and LDH assays, Figure 4C,D) compared to its effects on HT-29 cells (Figure 4A,B). This differential sensitivity may reflect variations in the mechanisms through which FU6 affects normal versus tumor cells, HEK293 cells being more susceptible to mitochondrial disruption and membrane damage. The discrepancies between the MTT and LDH results for HEK293 cells likely stem from the different mechanisms these assays assess. The MTT assay measures metabolic activity by detecting the mitochondrial reduction in MTT to formazan, while the LDH assay measures extracellular LDH release as an indicator of membrane damage. FU6 and FU13 were significantly more cytotoxic to HEK293 cells than to HT-29 cells (*p* < 0.05 for MTT; *p* < 0.0001 for LDH with FU6, and *p* < 0.01 for LDH with FU13) (Figure 4E,G). At 24 h, FU6 was more cytotoxic to HEK293 cells than to HT-29 cells based on MTT results (*p* < 0.05, Figure 4F), though this was not corroborated by LDH release (*p* < 0.0001). For FU13, at 24 h, a significantly higher LDH release was observed for HT-29 cells (*p* < 0.01), an effect confirmed by the MTT assay (Figure 4H). At 4 h, the effects of FU6 and FU13 were less pronounced, particularly for the HT-29 cell line, as indicated by both MTT and LDH assays. This suggests that significant cytotoxicity had not yet occurred, but the effects became more evident at 24 h. This difference may be attributed to the producer strains used in each formulation. FU6 contains metabolites produced by *Lactococcus lactis* UTNGt28 harboring genes encoding for multiple bacteriocins of sactipeptides class and enterolysin_A (unpublished), combined with exopolysaccharides (EPS) from *Lactiplantibacillus plantarum* UTNGt2. Functional genome annotation of UTNGt2 using Prokka identified genes encoding polysaccharide biosynthesis proteins, including *ywqE*, *murJ_1*, *murJ_2*, and capsular polysaccharide phosphotransferase cps12A. Additionally, several hypothetical proteins potentially involved in polysaccharide biosynthesis were detected. In contrast, FU13 was found to contain metabolites derived exclusively from *Weissella* [21]. Previous studies have similarly demonstrated that the cytotoxic effects of postbiotics can vary based on the producer strain. For instance, postbiotics from various *L. plantarum* strains exhibited greater cytotoxicity against HT-29 cancer cells, with IC50 values ranging from 22% to 28% (*v*/*v*) after 72 h of incubation [29]. Additionally, strain-dependent variations in cytotoxic effects on normal MCF-10A cell lines have been reported [29]. Consistent with prior research, our findings also corroborate the cytotoxic potential of MRS medium in a dose-dependent manner on human cells [33]. Nonetheless, the overall results indicate that FU6 and FU13 extracts did not exhibit significant cytotoxic effects on either normal or cancer cell lines at non-concentrated doses, underscoring their safety for potential applications. The time-dependent increase in cytotoxicity is likely due to the accumulation of damage over time, eventually overwhelming the cells’ protective mechanisms and resulting in mitochondrial dysfunction and membrane damage. Further research is warranted to explore the broader bioactive potential of these extracts.

### 2.4. Hemocompatibility

**Figure 5:**
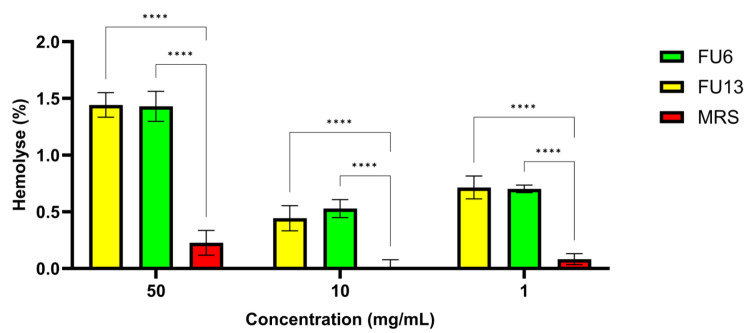
The hemolytic activity of erythrocyte suspensions is treated with different concentrations of samples. The results were presented as mean ± standard deviation from triplicate experiments. Statistical analysis using ordinary two-way ANOVA with Tukey’s multiple comparisons test showed significant differences between samples and culture media, indicated by **** *p* < 0.0001.

Erythrocytes are used as a prime candidate for the assessment of membranolytic or cytolytic activities, in addition to normal cell lines and primary cultured cells. Since significant cytotoxic effects were recorded for both FU6 and FU13 samples, at 1:1 and 1:2 dilutions, on normal and cancer cell lines, we performed a hemolysis study. Both FU6 and FU13 showed no hemolytic (<5%), while the TweenX-100 control showed a total blood lysis (Figure 5). The hemolysis index varies with the PBEs concentration, from 0.3 to 1.2%. Since a maximum of 5% hemolysis is allowed for biomaterials, the FU6 and FU13 postbiotics are safe as no negative effects on the permeability characteristics of membranes was detected. These results were in line with previous research findings demonstrating the metabolites from different *L. plantarum* species have no hemolytic effect [34]. The samples proved to be non-hemolytic, the differences between FU6 and FU13 being statistically insignificant (*p* > 0.05) for all concentrations considered. These results suggest that FU6 and FU13 do not disrupt red blood cells, indicating their low cytotoxicity.

### 2.5. Bioactive Molecules Composition of PBEs Revealed by Infrared Spectrometry

**Figure 6:**
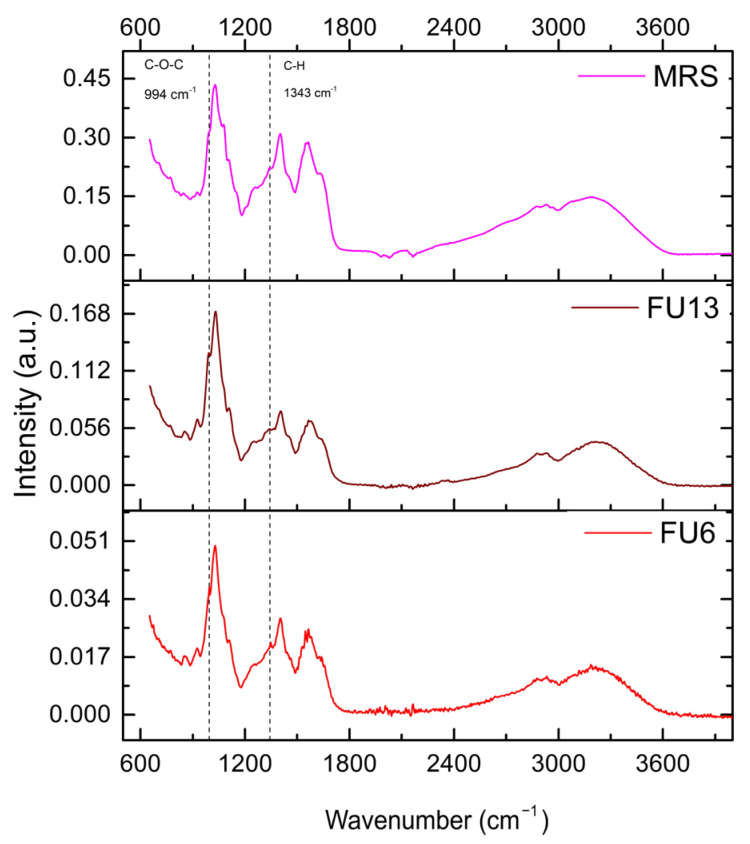
FTIR spectra of PBEs. Legend: MRS: MRS broth; FU6: CFS of UTNGt28 and EPS of UTNGt2 in proportion 3:1 (*v*/*v*); FU13: CFS of UTNGt21O. CFS: cell-free supernatant; EPS: exopolysaccharides.

For the uninoculated MRS medium, distinct spectral bands were identified, i.e.,: (i) 2920–2936 cm^−1^, associated with C-H stretching in lipids or carbohydrates, (ii) 1400–1566 cm^−1^, attributed to carboxyl or amide groups from peptones or nitrogenous compounds, and (iii) 1030–1108 cm^−1^, linked to polysaccharide or phosphate vibrations, present in both MRS and the FU6 and FU13 samples. Figure 6 illustrates the adsorption spectra. A broad peak near 3200 cm^−1^ indicates -OH stretching, while 2930 cm^−1^ corresponds to C-H stretching typical of polysaccharides. Intense bands at 1650–1550 cm^−1^ represent C=O and carboxyl vibrations. In the fingerprint region, peaks at 1406 cm^−1^ (C-H stretching), 1200–1000 cm^−1^ (C-O-C glycosidic bonds, C-O-H groups, and C-O stretching), and 925 cm^−1^ (glycosidic bonds) were observed. The 852–860 cm^−1^ range corresponds to bacterial polysaccharide -(1→3),(1→6)-α-D-glucan. Notably, bands at 995 cm^−1^ and 1343 cm^−1^, absent in the MRS medium, were specific to the EPS of Gt2, with FU6 showing both bands and FU13 displaying only the 995 cm^−1^ band. The 990–1000 cm^−1^ band corresponds to C-O-C vibrations in polysaccharides, indicating EPS presence, and the 1343 cm^−1^ band relates to C-H bending or carboxyl structures [35]. The absence of these bands in MRS suggests they are exclusive to bacterial EPS. Notably, the 996 cm^−1^ band, specific to glucose, requires a sufficient concentration of glycosidic residues in polysaccharides to enhance detection. These results confirm the unique molecular signatures of the EPS produced by lactic acid bacteria [36]. Further analyses are required to detect the compounds released in both CFS and EPS.

### 2.6. ^1^H-NMR Spectrum of CFS and EPS

**Figure 7:**
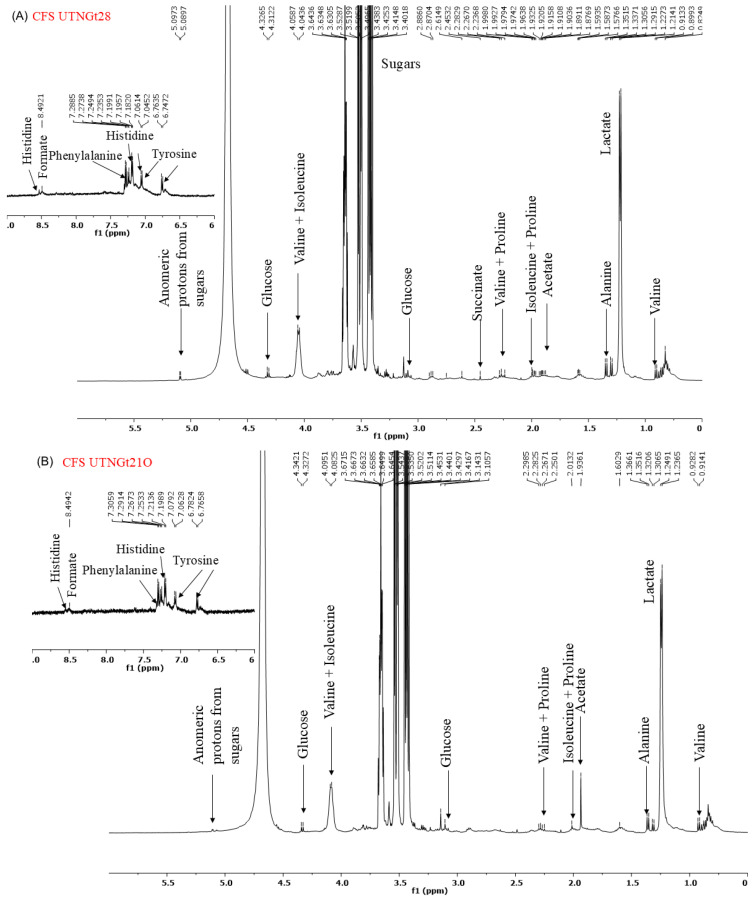

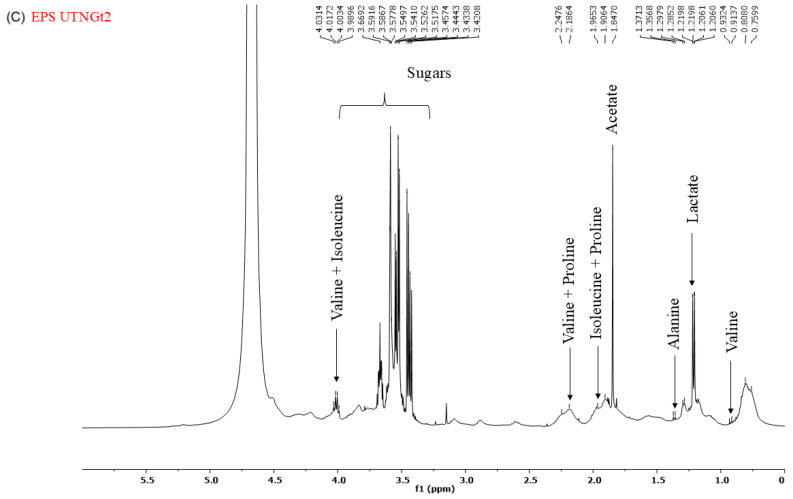
^1^H-NMR spectrum of (**A**). CFS UTNGt28; (**B**) CFS UTNGt21O; (**C**) CFS UTNGt2.

Analysis of the ^1^H-NMR spectra of the cell-free supernatant (CFS) and exopolysaccharides (EPS) (Figure 7A–C) revealed distinct metabolite profiles. The spectra obtained from the CFS of Gt28 and Gt21O strains exhibited peaks in the 7–9 ppm region, corresponding to aromatic and amide protons, and in the 3–5 ppm region, associated with hydroxyl and methoxy groups. These chemical shifts are characteristic of bioactive metabolites, including phenolics, peptides, and polyketides, which are commonly linked to antimicrobial activity. In contrast, the EPS from the *Gt2* strain lacked functional groups typically involved in antimicrobial mechanisms. Instead, its NMR spectra were dominated by carbohydrate-like signals within the 3.0–5.5 ppm range, including prominent anomeric proton peaks (~4.5–5.5 ppm) and broad hydroxyl signals (~3–4 ppm), indicative of a polysaccharide-based structure. Such structural characteristics suggest a role in biofilm formation or immune modulation rather than direct antimicrobial activity. Furthermore, the absence of amide (-NH, ~7–9 ppm) and carboxyl (-COOH, ~10–12 ppm) groups further supports the lack of bactericidal properties. These findings suggest that the EPS produced by Gt2 primarily contributes to adhesion and microbial interactions rather than exerting direct antimicrobial effects.

### 2.7. Antioxidant Activity of PBEs

**Figure 8:**
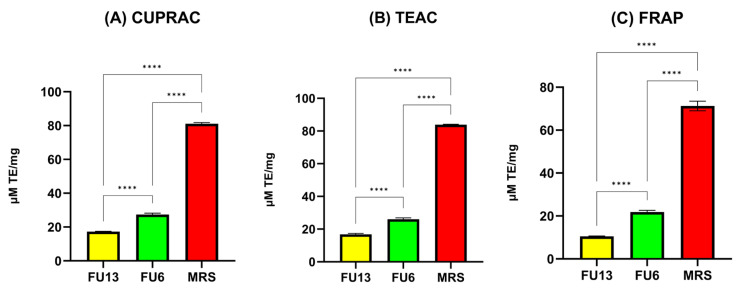
Antioxidant activity of PBEs (**A**) CUPRAC, (**B**) TEAC and (**C**) FRAP assays. FU6: CFS of UTNGt28 and EPS of UTNGt2 in proportion 3:1 (*v*/*v*); FU13: CFS of UTNGt21O. CFS: cell-free supernatant; EPS: exopolysaccharides; MRS: MRS broth. Statistical analysis using ordinary one-way ANOVA with Tukey’s multiple comparisons test showed significant differences between samples and culture media, indicated by **** *p* < 0.0001.

**Figure 9:**
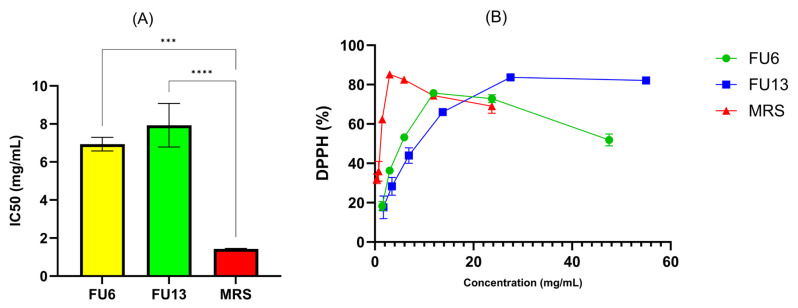
Antioxidative activities of PBEs expressed as (**A**) IC50 (mg/mL) and (**B**) DPPH free radical scavenging activity (%) for each concentration. FU6: CFS of UTNGt28 and EPS of UTNGt2 in proportion 3:1 (*v*/*v*); FU13: CFS of UTNGt21O. CFS: cell-free supernatant; EPS: exopolysaccharides; MRS: MRS broth. Statistical analysis using ordinary one-way ANOVA with Tukey’s multiple comparisons test showed significant differences between samples and culture media, indicated by *** *p* < 0.001 and **** *p* < 0.0001.

LAB strains are gaining attention for their role in promoting human health by reducing oxidative stress (ROS), epidemiological studies linking lactobacilli to a lower risk of chronic diseases, with benefits attributed to both viable cells and CFS [37]. In our study, the antioxidant activity results measured by the CUPRAC, TEAC, and FRAP methods highlight key differences in the antioxidant properties of MRS broth and FU6 and FU13 extracts. The CUPRAC and TEAC methods (Figure 8) showed similar trends in antioxidant activity. FU6 exhibited significantly higher antioxidant activity than FU13 (*p* < 0.0001), suggesting the presence of more efficient or higher concentrations of antioxidant compounds. The FRAP method (Figure 8) yielded lower absolute values for antioxidant activity compared to the other two methods, but the trends remained consistent: MRS (known to be rich in antioxidant compounds) > FU6 > FU13. The lower values from the FRAP method may be due to its reliance on the reduction in ferric ions (Fe^3^⁺) to ferrous ions (Fe^2^⁺), which might be less sensitive to certain antioxidants present in the EPS. The lower antioxidant activity observed in FU6 and FU13 which are a combination of metabolites released in the cell free supernatant and exopolysaccharides produced by lactic acid bacteria, reflects the consumption and transformation of the antioxidant species during bacterial metabolism. The higher activity of FU6 compared to FU13 suggests strain-specific differences in metabolic processes and antioxidant activity. Moreover, using the DPPH method (Figure 9), the IC50 values were determined within the concentration range of 11.90–0.74 mg/mL for FU6, which reached a maximum of 75.74 ± 0.23%. For FU13, the linearity range was 27.5–1.72 mg/mL, with a maximum value at 83.72 ± 0.40%. These IC50 results are consistent with the trends observed using the CUPRAC, FRAP, and TEAC methods. For the MRS medium, the linearity range was narrower, between 2.96 and 0.19 mg/mL, with a maximum value of 85.23 ± 0.65%. While the antioxidant effects among FU6, FU13, and MRS were not significantly different in the DPPH assay, the differences compared to the culture medium were statistically significant (FU6 vs. MRS: *p* < 0.001, FU13 vs. MRS: *p* < 0.0001). This suggests that although FU6 and FU13 demonstrate notable antioxidant activity, their efficacy remains lower than that of the MRS medium, likely due to the consumption or transformation of antioxidant compounds during bacterial metabolism. However, these results agree with other studies showing that CFS is recognized as a rich source of antioxidants [38]. This activity is likely linked to metabolites secreted in CFS, including tartaric acid, phenyl lactic acid, lactic acid, linoleic acid, pyruvic acid, maleic acid, homovanillic acid, xylose, fumaric acid, glyceraldehyde, and hydroxyphenyl lactic acid [39].

## 3. Materials and Methods

### 3.1. Bacterial Strains and Growth Conditions

The LAB strains: *Lactiplantibacillus plantarum* UTNGt2 (GenBank Genome Accession SRX10182863), *Lactococcus lactis* UTNGt28 (GenBank accession No. MG675576.1) and *Weissella cibaria* UTNGt21O (GenBank Genome Assembly SRX8614718) previously isolated and characterized were routinely grown in MRS broth (Difco, Detroit, MI, USA). The multidrug resistant *E. coli* L1PEag1 strain (GenBank Genome Assembly GCF_036870985.1) was grown in Luria–Bertani (Difco, Detroit, MI, USA) broth culture media. The chemicals and reagents used were of analytical grade.

### 3.2. Extraction of CFS and EPS, and Selection of PBEs with High Antimicrobial Activity Against E. coli L1PEag1

The LAB strains were grown in MRS broth at 37 °C for 27 h, then CFS was separated by centrifugation at 13,000× *g* for 30 min (4 °C). The CFS was then filtered using 0.22 µm porosity syringe filter (a microporous filter made of polytetrafluoroethylene (PTFE) membrane) and stored at 4 °C for further analysis (#STF020025H, Chemlab Group, Washington, DC, USA). EPS was extracted by centrifuging a 24 h bacterial culture (1 × 10^8^ CFU/mL) grown in MRS medium supplemented with 20% sucrose (MRSS) at 10,000× *g* for 20 min at 4 °C followed by adding two volumes of ice-cold absolute ethanol and refrigerating the mixture for two days. The precipitated EPS was centrifuged, dissolved in double-distilled water, and dialyzed using a midi dialysis kit (cat # PURD10005-1KT, Sigma-Aldrich, St. Louis, MO, USA). The final products, CFS and EPS, were lyophilized before use.

### 3.3. Characterization of Selected PBEs

Table S1 describes all the extracts that were tested for their antimicrobial activity against *E. coli* L1PEag1 [16]. The following extracts, (1) FU6, consisting of CFS from UTNGt28 and EPS from UTNGt2 in proportion 3:1 (*v*/*v*) (final concentration 95 mg/mL) and (2) FU13, consisting of CFS from UTNGt21O (final concentration 100 mg/mL), were further characterized. For the antimicrobial assay two controls, C1: MRS broth and C2: MRSS broth were used.

#### 3.3.1. Antimicrobial Activity

##### Determination of Minimum Inhibitory Concentration (MIC)

MIC determination was performed as previously described [40]. In brief, FU6 and FU13 samples were tested in serial two-fold dilution gradients in LB broth medium seeded with after indicator bacteria *E. coli* L1PEag1 at 1 × 10^6^ CFU/mL density. The control groups were blank medium and sterile phosphate-buffered saline (PBS) solution (pH 7.2). The tubes were then incubated for 18 h at 37 °C, and then known volumes were spotted on plate agar to determine the MIC that reduced the bacterial growth by 90% [41]. Each sample was set up in three replicates and the assays were carried out three times independently.

##### Time-Killing Assay

The time-kill experiments were conducted as previously described [16]. *E. coli* L1PEag1 (1 × 10^6^ CFU/mL) was inoculated independently with FU6 and FU13 at 0.25 × MIC, 0.5 × MIC, and 1 × MIC concentrations and incubated at 37 °C for 18 h. An untreated cell culture was used as a control. The plate agar method (BD Difco plate count agar, Fisher Scientific Co. LLC, Hampton, NH, USA) was used to determine the viability of the cells at various time intervals of 0, 2, 3, 5, 7, and 18 h. By calculating the difference between the log10 (CFU) of the untreated cells (those lacking FU6 or FU13) and the treated cells (those with FU6 or FU13), the results were analyzed to determine the percentage of reduction. The most significant cell reduction (*p* < 0.05) was defined as >90% [42]. All experiments were performed in triplicate.

##### The Examination of Ultrastructural and Morphological Changes in E. coli L1PEag1 upon PBEs Treatment

Following the previously described protocol, L1PEag1 cell culture in exponential phase (1 × 10^6^ CFU/mL) was treated with 0.5 × MIC of FU6, FU13, EPS of Gt2 and CFS of Gt28 at 0.5 × MIC for 18 h at 37 °C. The cells were fixed with 2.5% glutaraldehyde and stored overnight at 4 °C then washed three times with cacodylate buffer, postfixed for 1 h in 1% osmium tetroxide (1:1 with cacodylate buffer), and incubated overnight in the same buffer after three additional washes. Further, they were washed with water, treated with uranyl acetate (Sigma-Aldrich Co. LLC, Saint Louis, MO, USA), washed again, dehydrated in a graded ethanol series, and embedded in Epon resin. Ultrathin sections were mounted on copper grids, stained with uranyl acetate and lead citrate (Sigma-Aldrich Co. LLC, Saint Louis, MO, USA), and analyzed using a Tecnai G2 F20 transmission electron microscope (FEI Company, Hillsboro, OR, USA). For SEM analysis, the samples were put on graphite tape. Each sample was covered with a thin layer of gold, about 24.5 nm thick, using DENTON VACUUM Desk IV equipment (DENTON VACUUM, Austin, TX, USA). To create high-resolution images, the samples were subsequently inspected using a high vacuum scanning electron microscope. Using the secondary electron detector, the samples’ topography and morphology were investigated. The samples were examined using a JSM-6490 LV Scanning Electronic Microscopy apparatus (JEOL, JSM, Peabody, MA, USA).

#### 3.3.2. Biocompatibility Assessment

Human embryonic kidney cells (HEK293, CRL-1573 line) (2 × 10^4^ cells/well) and goblet-like mucus-producing intestinal cells (HT-29-MTX line) (4 × 10^4^ cells/well) [43] were used for cytotoxicity testing, using 96-well plates and Dulbecco’s Modified Eagle Medium supplemented with 10% fetal bovine serum. After adhesion, the cells were incubated for 4 or 24 h with the tested samples at 37 °C, 95% humidity with 5% CO_2_.

##### Cells Viability Determination: MTT Assay

The MTT (3-(4,5-dimethylthiazol-2-yl)-2,5-diphenyltetrazolium) (Sigma-Aldrich Inc., St. Louis, MO, USA) assay is a colorimetric test that provides data on cell viability [44]. The test is based on the principle that metabolically active cells can reduce the yellow tetrazolium salt MTT by brown mitochondrial dehydrogenases (P) H-dependent. The MTT compound can pass through the living cell membranes, being broken down into soluble formazan crystals. After 4 and 24 h of incubation of the culture with the tested suspensions, the culture medium was removed from each well, and 130 µL of MTT solution (1 mg/mL concentration) were added. After 90 min of incubation at 37 °C and 5% CO_2_, the MTT solution was aspirated and the formazan crystals in each well were solubilized with 130 µL of 2-propanol per well. The absorbance was measured at 595 nm using a FlexStation 3 UV-VIS spectrophotometer (Molecular Devices Company, Sunnyvale, CA, USA), microplate reader.

##### Lactate Dehydrogenase (LDH) Release Assay

LDH is a widely used method for assessing cell damage and cytotoxicity, as LDH is released into the extracellular environment when cell membranes are compromised [45]. The assay quantifies LDH activity through the reduction in NAD⁺ to NADH by cytosolic LDH, which occurs when LDH is released in the culture medium. This reaction converts lactate to pyruvate while reducing NAD⁺ to NADH, which subsequently reduces INT (a tetrazolium salt) to produce a red formazan product. Following a 4 and 24 h incubation period with FU6 and FU13, the culture medium was removed, and LDH release was determined in accordance with the manufacturer’s instructions using the Cytotoxicity Detection Kit PLUS (Roche, NY, USA). Volumes of 50 μL culture supernatants were mixed with 50 μL catalyst and dye solution reaction mixture, and the mix was incubated for 30 min in a dark environment. Using a microplate reader (FlexStation 3, Molecular Devices Company, Sunnyvale, CA, USA), the absorbance was measured at 490 nm after the reaction was stopped with 50 μL of stop solution.

##### Hemolytic Effect on Red Blood Cells

Hemolysis assay was performed on sheep red blood cells as previously described [46]. Briefly, to prevent blood clotting, a volume of 9 mL of the blood sample was suspended in 1 mL of 10% citric acid dextrose and the tube was centrifuged at 5000 rpm for 10 min. The supernatant was removed, and the pellet containing red blood cells was washed thrice with PBS (0.2 M, pH 7.4) before resuspension in sterile saline solution (0.9%). Furthermore, a volume of 100 µL of the FU6 and FU13 at varying dilutions (50 mg/mL, 10 mg/mL, and 1 mg/mL) was dispensed into 400 µL of erythrocyte suspension (10%). The mixtures were incubated 1 h at 37 °C. The samples were centrifuged at 5000 rpm for 10 min, and the absorbance of the supernatant was measured at 540 nm. The same amount of erythrocyte suspension was added to Triton × 100 (1%) and PBS 1×, respectively, to obtain the positive and negative controls. The samples were incubated, then centrifuged for 10 min at 5000 rpm. The supernatant was distributed in 96-well plates and the absorbance of the supernatant at 540 nm using a FlexStation 3 UV-VIS spectrophotometer (Molecular Devices Company, Sunnyvale, CA, USA). The hemolytic index (HI%) percentage was determined in accordance with Marinas et al. [46].

#### 3.3.3. ATR-FTIR Assay

FTIR spectra for all freeze-dried samples were obtained using a Cary 630 FTIR Spectrometer in ATR mode, in conjunction with the Agilent MicroLab Software FTIR System v5.5.1989 (Agilent Technologies, Inc., Santa Clara, CA, USA). The measurements were performed across a spectral range of 4000–650 cm^−1^, utilizing 400 scans with a resolution of 4 cm^−1^ at room temperature in air. FTIR scanning was performed on the lyophilized MRS medium and FU6 and FU13 samples.

#### 3.3.4. ^1^H-NMR Assay

A 10 mg lyophilized sample of CFS UTNGt28, CFS Gt21O, and EPS UTNGt2 was dissolved in 1 mL of D2O (99.8% D) and placed in a 0.5 mm NMR tube (Norell NOR508UP7-5EA). NMR spectra were obtained using a Bruker Avance III Ultrashield Plus spectrometer operating at 11.74 T, with a resonance frequency of 500.13 MHz for the ^1^H nucleus. Chemical shifts (δ) were referenced to residual solvent peaks. The ^1^H-NMR experiments were conducted with the following parameters: pulse program zg30, 8.3 s acquisition time, 8.01 kHz spectral window, 64 scans (ns), 20 K data points, and a 2 s delay time. For ^1^H-NMR solvent suppression, NOESY sequence experiments were carried out with the parameters: pulse program noesyphpr, 3.3 s acquisition time, 10 kHz spectral window, 64 scans (ns), 20 K data points, 5 s delay time (d1), and a 200 ms mixing time (d8).

#### 3.3.5. Antioxidant Activity

##### DPPH (1,1-Diphenyl-2-picrylhydrazyl) Assay

The free radical scavenging activity of FU6 and FU13 formulation was evaluated using a spectrophotometric method described by Madhu et al. [47]. A 0.3 mM DPPH solution was prepared in methanol and vortexed until fully dissolved. FU6 and FU13 extracts at various concentrations (0.75–50 mg of dry extract/mL in deionized water) were prepared. Then, 200 µL of each extract concentration was mixed with 200 µL of the DPPH solution. The mixture was incubated in the dark for 20 min, followed by centrifugation at 5000 rpm for 10 min. Absorbance was measured at 517 nm using a spectrophotometer. All experiments were performed in triplicate. The percentage of DPPH inhibition was calculated using the following formula: DPPH inhibition (%) = A − B/A × 100, where A represents the absorbance of the solvent control (control—DPPH) and B denotes the absorbance of PBEs. The concentration needed to achieve 50% inhibition of the DPPH radical (IC50) was determined using linear regression analysis of concentration versus percentage of DPPH inhibition. The data were further analyzed using the log(inhibitor) versus response—variable slope (four-parameter) model in Prism GraphPad 10.0 software.

##### CUPRAC (Cupric Reducing Antioxidant Capacity) Assay

The CUPRAC method is based on the reduction in a cupric–neocuproine complex by antioxidants [48]. In brief, 60 µL of each sample or standard solution at various concentrations was mixed with 50 µL of CuCl₂ (10 mM), 50 µL of neocuproine (7.5 mM), and 50 µL of 1 M ammonium acetate buffer (pH 7.0). After a 20 min incubation, the samples were centrifuged for 10 min at 5000 rpm, and absorbance was recorded at 450 nm. Trolox stock solutions (2 mM) were utilized to generate a calibration curve with working concentrations ranging from 0.125 to 2.0 mM (R^2^ = 0.9976). The results were expressed as mM Trolox equivalents per gram of dry extract.

##### FRAP (Ferric Reducing Antioxidant Power) Assay

In the FRAP assay, 285 µL of the FRAP reagent, was added to 15 µL of the sample [49]. The reaction mixture was incubated in the dark at 37 °C for 25 min, after which it was centrifuged (Thermo Scientific, Waltham, MA, USA). Absorbance was measured at 593 nm using a FlexStation 3 UV–VIS spectrophotometer (Molecular Devices, Sunnyvale, CA, USA). A Trolox calibration curve (20–250 µM) was established, yielding an R^2^ value of 0.9991.

##### TEAC (Trolox Equivalent Antioxidant Capacity) Assay

The Trolox Equivalent Antioxidant Capacity (TEAC) assay was conducted according to the method outlined by Re et al. [50] with minor modifications. A stable ABTS⁺ stock solution was prepared by mixing 7 mM ABTS with 2.45 mM potassium persulfate, allowing the mixture to stand in the dark at room temperature for 12–16 h. The ABTS⁺ working solution was obtained by diluting the stock with ethanol to achieve an absorbance of approximately 0.70. For the assay, 60 µL of the sample or standard was combined with 540 µL of the ABTS⁺ working solution and incubated in the dark for 20 min before centrifugation at 5000 rpm for 10 min. A standard curve was generated using Trolox concentrations ranging from 20 to 200 µM (R^2^ = 0.997). The results were reported as mM Trolox equivalents per gram of dry sample.

### 3.4. Statistical Analysis

By using triplicate analysis, the means ± SD of the data were reported. To perform the statistical analysis, GraphPad Prism v10 (GraphPad Software, San Diego, CA, USA) was used. A one-way ANOVA was applied to assess antioxidant activity, and Tukey’s method was used for multiple comparison corrections, employing a single pooled variance to evaluate the effects of metabolic extracts and culture media (MRS). Statistical significance was determined at a threshold of *p* < 0.05. Individual variances were computed for comparisons between samples (FU6 and FU13) and the positive control (microbial strain) for qualitative and quantitative antimicrobial activities.

## 4. Conclusions

These findings underscore the antimicrobial efficacy of PBEs, particularly FU6 and FU13, against the multidrug-resistant *E. coli* L1PEag1 strain. Notably, FU6 exhibited sustained inhibition for 18 h, while both extracts demonstrated a rapid bactericidal effect at 1× MIC, highlighting their strong and time-dependent activity. Morphological and ultrastructural analyses confirmed severe membrane damage, leading to cell rupture and intracellular leakage, indicating a direct disruption of bacterial integrity and ultimate cell death. Crucially, cytotoxicity assessments confirmed that while both extracts exhibit time-dependent effects, their toxicity remains within acceptable limits, supporting their potential for food applications. Hemocompatibility tests further validated their safety, as neither FU6 nor FU13 induced hemolysis. Spectroscopic analysis identified key bioactive compounds, including exopolysaccharides and bacteriocins, which likely contribute to their potent antimicrobial properties. Overall, these results establish PBEs as promising multifunctional antimicrobial agents capable of effectively inhibiting foodborne pathogens while maintaining biocompatibility. Their ability to disrupt bacterial membranes and cause structural damage highlights their potential for food preservation, enhancing food safety, and extending shelf life. Future research should delve into their molecular mechanisms of action and validate their efficacy in real food systems. Challenge tests in postharvest fruits will assess the stability and persistence of PBEs under storage conditions, while in vivo contamination models will simulate real-world scenarios to confirm their protective effects. Additionally, food matrix compatibility studies are essential to determine how PBEs interact with proteins, lipids, and carbohydrates, ensuring their stability and functionality in complex environments. Investigating factors such as pH, moisture content, and fat composition will provide deeper insights into their antimicrobial performance. Finally, sensory analysis will be necessary to assess any impact on the organoleptic properties of treated foods. These comprehensive evaluations will help establish PBEs as effective and safe antimicrobial agents for food preservation, ensuring their practical applicability in maintaining food quality and safety.

## Data Availability

The original contributions presented in this study are included in the article/Supplementary Materials. Further inquiries can be directed to the corresponding author.

## Supplementary Materials

The following supporting information can be downloaded at: https://www.mdpi.com/article/10.3390/antibiotics14030318/s1, Table S1: Formulation description and antimicrobial activity against *Escherichia coli* L1PEag1; Table S2. The MIC values against indicator pathogenic bacteria *E. coli* L1PEag1.

## References

[1] Wang Y., Wu J., Lv M., Shao Z., Hungwe M., Wang J., Bai X., Xie J., Wang Y., Geng W. (2021). Metabolism characteristics of lactic acid bacteria and the expanding applications in food industry. Front. Bioeng. Biotechnol..

[2] Isaac-Bamgboye F.J., Mgbechidinma C.L., Onyeaka H., Isaac-Bamgboye I.T., Chukwugozie D.C. (2024). Exploring the potential of postbiotics for food safety and human health improvement. J. Nutr. Metab..

[3] Vera-Santander V.E., Hernández-Figueroa R.H., Jiménez-Munguía M.T., Mani-López E., López-Malo A. (2023). Health benefits of consuming foods with bacterial probiotics, postbiotics, and their metabolites: A review. Molecules.

[4] Thorakkattu P., Khanashyam A.C., Shah K., Babu K.S., Mundanat A.S., Deliephan A., Deokar G.S., Santivarangkna C., Nirmal N.P. (2022). Postbiotics: Current trends in food and pharmaceutical industry. Foods.

[5] Scott E., De Paepe K., Van de Wiele T. (2022). Postbiotics and Their Health Modulatory Biomolecules. Biomolecules.

[6] Salminen S., Collado M.C., Endo A., Hill C., Lebeer S., Quigley E.M.M., Sanders M.E., Shamir R., Swann J.R., Szajewska H. (2021). The International Scientific Association of Probiotics and Prebiotics (ISAPP) consensus statement on the definition and scope of postbiotics. Nat. Rev. Gastroenterol. Hepatol..

[7] Tumbarski Y., Nikolova R., Petkova N., Ivanov I., Lante A. (2019). Biopreservation of fresh strawberries by carboxymethyl cellulose edible coatings enriched with a bacteriocin from *Bacillus methylotrophicus* BM47. Food Technol. Biotechnol..

[8] Vieira A.I., Guerreiro A., Antunes M.D., Miguel MD G., Faleiro M.L. (2019). Edible coatings enriched with essential oils on apples impair the survival of bacterial pathogens through a simulated gastrointestinal system. Foods.

[9] Tsilingiri K., Barbosa T., Penna G., Caprioli F., Sonzogni A., Viale G., Rescigno M. (2012). Probiotic and postbiotic activity in health and disease: Comparison on a novel polarised ex-vivo organ culture model. Gut.

[10] Cicenia A., Scirocco A., Carabotti M., Pallotta L., Marignani M., Severi C. (2014). Postbiotic activities of lactobacilli-derived factors. J. Clin. Gastroenterol..

[11] Rajoka M.S.R., Zhao H., Lu Y., Lian Z., Li N., Hussain N., Shao D., Jin M., Li Q., Shi J. (2018). Anticancer potential against cervix cancer (HeLa) cell line of probiotic *Lactobacillus casei* and *Lactobacillus paracasei* strains isolated from human breast milk. Food Funct..

[12] Islam S., Biswas S., Jabin T., Moniruzzaman M., Biswas J., Uddin M.S., Akhtar-E-Ekram M., Elgorban A.M., Ghodake G., Syed A. (2023). Probiotic potential of *Lactobacillus plantarum* DMR14 for preserving and extending shelf life of fruits and fruit juice. Heliyon.

[13] Biswas I., Das Mohapatra P.K. (2023). Recent advancement in metabiotics: A consortium with bioactive molecules after fermentation by probiotic bacteria with multidisciplinary application potential and future solution in health sector. Bioresour. Technol. Rep..

[14] Vieco-Saiz N., Belguesmia Y., Raspoet R., Auclair E., Gancel F., Kempf I., Drider D. (2019). Benefits and inputs from lactic acid bacteria and their bacteriocins as alternatives to antibiotic growth promoters during food-animal production. Front. Microbiol..

[15] Tenea G.N. (2021). Postbiotics: A solution to protect tropical fruits towards postharvest adulteration. AgroLife Sci. J..

[16] Tenea G.N., Angamarca E., Olmedo D. (2022). Combinations of peptide-protein extracts from native probiotics suppress the growth of multidrug-resistant *Staphylococcus aureus* and *Citrobacter freundii* via membrane perturbation and ultrastructural changes. Antibiotics.

[17] Tenea G.N., Angamarca E., Cifuentes V., Hidalgo J. (2024). Preventing microbe colonization on avocado (*Persea nubigena* var. guatemalensis) through metabiotic treatment, a promising postharvest safety improvement. Front. Microbiol..

[18] Molina D., Carrión-Olmedo J.C., Jarrín-V P., Tenea G.N. (2024). Genome characterization of a multi-drug resistant *Escherichia coli* strain, L1PEag1, isolated from commercial cape gooseberry fruits (*Physalis peruviana* L.). Front. Microbiol..

[19] Rocchetti M.T., Russo P., Capozzi V., Drider D., Spano G., Fiocco D. (2021). Bioprospecting antimicrobials from *Lactiplantibacillus plantarum*: Key factors underlying its probiotic action. Int. J. Mol. Sci..

[20] Kang C.E., Park Y.J., Kim J.H., Lee N.K., Paik H.D. (2023). Probiotic *Weissella cibaria* displays antibacterial and anti-biofilm effect against cavity-causing *Streptococcus mutans*. Microb. Pathog..

[21] Tenea G.N., Hurtado P. (2021). Next-generation sequencing for whole-genome characterization of *Weissella cibaria* UTNGt21O strain originated from wild *Solanum quitoense* Lam. fruits: An atlas of metabolites with biotechnological significance. Front. Microbiol..

[22] Remus D.M., van Kranenburg R., van Swam I.I., Taverne N., Bongers R.S., Wels M., Wells J.M., Bron P.A., Kleerebezem M. (2012). Impact of 4 *Lactobacillus plantarum* capsular polysaccharide clusters on surface glycan composition and host cell signaling. Microb. Cell Fact..

[23] Abdalla A.K., Ayyash M.M., Olaimat A.N., Osaili T.M., Al-Nabulsi A.A., Shah N.P., Holley R. (2021). Exopolysaccharides as antimicrobial agents: Mechanism and spectrum of activity. Front. Microbiol..

[24] Cushnie T.P., Lamb A.J. (2005). Antimicrobial activity of flavonoids. Int. J. Antimicrob. Agents.

[25] Wang Z., Liu X., Teng D., Mao R., Hao Y., Yang N., Wang X., Li Z., Wang X., Wang J. (2020). Development of chimeric peptides to facilitate the neutralization of lipopolysaccharides during bactericidal targeting of multidrug-resistant *Escherichia coli*. Commun. Biol..

[26] Wang G., Zeng H. (2022). Antibacterial effect of cell-free supernatant from *Lactobacillus pentosus* L-36 against *Staphylococcus aureus* from bovine mastitis. Molecules.

[27] Ozma M.A., Ghotaslou R., Asgharzadeh M., Abbasi A., Rezaee M.A., Kafil H.S. (2024). Cytotoxicity assessment and antimicrobial effects of cell-free supernatants from probiotic lactic acid bacteria and yeast against multidrug-resistant *Escherichia coli*. Lett. Appl. Microbiol..

[28] Westmoreland C., Holmes A.M. (2009). Assuring consumer safety without animals: Applications for tissue engineering. Organogenesis.

[29] Chuah L.O., Foo H.L., Loh T.C., Mohammed Alitheen N.B., Yeap S.K., Abdul Mutalib N.E., Abdul Rahim R., Yusoff K. (2019). Postbiotic metabolites produced by *Lactobacillus plantarum* strains exert selective cytotoxicity effects on cancer cells. BMC Complement. Altern. Med..

[30] Nandhra G.K., Mark E.B., Di Tanna G.L., Haase A., Poulsen J., Christodoulides S., Kung V., Klinge M.W., Knudsen K., Borghammer P. (2020). Normative values for region-specific colonic and gastrointestinal transit times in 111 healthy volunteers using the 3D-Transit electromagnet tracking system: Influence of age, gender, and body mass index. Neurogastroenterol. Motil..

[31] Procházková N., Falony G., Dragsted L.O., Licht T.R., Raes J., Roager H.M. (2023). Advancing human gut microbiota research by considering gut transit time. Gut.

[32] Roma D., Cecchini M.E., Tonini M.P., Capella V., Aiassa D., Rodriguez N., Mañas F. (2023). Toxicity assessment and DNA repair kinetics in HEK293 cells exposed to environmentally relevant concentrations of Glyphosate (Roundup^®^ Control Max). Toxicol. Res..

[33] Tan H.K., Foo H.L., Loh T.C., Banu N., Alitheen M., Rahim A.R. (2015). Cytotoxic effect of proteinaceous postbiotic metabolites produced by *Lactobacillus plantarum* I-UL4 cultivated in different media composition on MCF-7 breast cancer cell. Malays. J. Microbiol..

[34] Thirabunyanon M., Boonprasom P., Niamsup P. (2009). Probiotic potential of lactic acid bacteria isolated from fermented dairy milks on antiproliferation of colon cancer cells. Biotechnol. Lett..

[35] Cárdenas-Escudero J., Galán-Madruga D., Cáceres J.O. (2023). Rapid, reliable and easy-to-perform chemometric-less method for rice syrup adulterated honey detection using FTIR-ATR. Talanta.

[36] Arefi A., Sturm B., Babor M., Horf M., Hoffmann T., Höhne M., Friedrich K., Schroedter L., Venus J., Olszewska-Widdrat A. (2024). Digital model of biochemical reactions in lactic acid bacterial fermentation of simple glucose and biowaste substrates. Heliyon.

[37] Xing J., Wang G., Zhang Q., Liu X., Gu Z., Zhang H., Chen Y.Q., Chen W. (2015). Determining antioxidant activities of lactobacilli cell-free supernatants by cellular antioxidant assay: A comparison with traditional methods. PLoS ONE.

[38] Lee J., Hwang K.T., Heo M.S., Lee J.H., Park K.Y. (2005). Resistance of *Lactobacillus plantarum* KCTC 3099 from Kimchi to oxidative stress. J. Med. Food.

[39] Gu X., Wang H., Wang L., Zhang K., Tian Y., Wang X., Xu G., Guo Z., Ahmad S., Egide H. (2024). The antioxidant activity and metabolomic analysis of the supernatant of *Streptococcus alactolyticus* strain FGM. Sci. Rep..

[40] Xiang Y., Li X., Zheng H., Chen J.Y., Lin L.B., Zhang Q.L. (2021). Purification and antibacterial properties of a novel bacteriocin against *Escherichia coli* from *Bacillus subtilis* isolated from blueberry ferments. LWT-Food Sci. Technol..

[41] Yasir M., Dutta D., Willcox M.D.P. (2020). Activity of antimicrobial peptides and ciprofloxacin against *Pseudomonas aeruginosa* biofilms. Molecules.

[42] Ge J., Sun Y., Xin X., Wang Y., Ping W. (2016). Purification and partial characterization of a novel bacteriocin synthesized by *Lactobacillus paracasei* HD1-7 isolated from Chinese sauerkraut juice. Sci. Rep..

[43] Alemka A., Clyne M., Shanahan F., Tompkins T., Corcionivoschi N., Bourke B. (2010). Probiotic colonization of the adherent mucus layer of HT29MTXE12 cells attenuates *Campylobacter jejuni* virulence properties. Infect. Immun..

[44] van Meerloo J., Kaspers G.J., Cloos J. (2011). Cell sensitivity assays: The MTT assay. Methods Mol. Biol..

[45] Chan F.K., Moriwaki K., De Rosa M.J. (2013). Detection of necrosis by release of lactate dehydrogenase activity. Methods Mol. Biol..

[46] Marinas I.C., Ignat L., Maurușa I.E., Gaboreanu M.D., Adina C., Popa M., Chifiriuc M.C., Angheloiu M., Georgescu M., Iacobescu A. (2024). Insights into the physico-chemical and biological characterization of sodium lignosulfonate—Silver nanosystems designed for wound management. Heliyon.

[47] Madhu G., Bose V.C., Aiswaryaraj A.S., Maniammal K., Biju V. (2013). Defect dependent antioxidant activity of nanostructured nickel oxide synthesized through a novel chemical method. Colloids Surf. Physicochem. Eng. Asp..

[48] Celik S.E., Ozyürek M., Güçlü K., Apak R. (2010). Determination of antioxidants by a novel on-line HPLC-cupric reducing antioxidant capacity (CUPRAC) assay with post-column detection. Anal. Chim. Acta.

[49] Benzie I.F.F., Strain J.J. (1999). Ferric reducing/antioxidant power assay: Direct measure of total antioxidant activity of biological fluids and modified version for simultaneous measurement of total antioxidant power and ascorbic acid concentration. Methods Enzymol..

[50] Re R., Pellegrini N., Proteggente A., Pannala A., Yang M., Rice-Evans C. (1999). Antioxidant activity applying an improved ABTS radical cation decolorization assay. Free Radic. Biol. Med..

