# Supplementary material for: Postbiotic-Based Extracts from Native Probiotic Strains: A Promising Strategy for Food Preservation and Antimicrobial Defense
Source: Antibiotics (Basel). 2025 Mar 18;14(3):318. doi: 10.3390/antibiotics14030318 (PMC11939163; doi:10.3390/antibiotics14030318)
Supplement: Supplementary file 1 [file antibiotics-14-00318-s001.zip › antibiotics-3525895-supplementary.pdf]

antibiotics

Supplementary files

Postbiotic-Based Extracts from Native Probiotic Strains: A Promising Strategy for Food Preservation and Antimicrobial Defense

Diana Molina [1], Ioana C. Marinas [2], Evelyn Angamarca [1], Anamaria Hanganu [3,4], Miruna Stan [2], Mariana C. Chifiriuc [2] and Gabriela N. Tenea [1*]

[1]Biofood and Nutraceutics Research and Development Group; Faculty of Engineering in Agricultural and Environmental Sciences, Universidad Técnica del Norte. Av. 17 de Julio s-21 y José María Córdova. 100150, Ibarra, Ecuador.
[2] Research Institute of the University of Bucharest—ICUB, 91-95 Splaiul Independentei St., District 5,050095 Bucharest, Romania
[3] Department of Inorganic and Organic Chemistry, Biochemistry and Catalysis, Faculty of Chemistry, University of Bucharest, RO-050663 Bucharest, Romania
[4]"C. D. Nenitzescu" Institute of Organic and Supramolecular Chemistry of the Romanian Academy, 060023 Bucharest, Romania

* Corresponding author: gntenea@utn.edu.ec

Table S1. Formulation description and antimicrobial activity against *Escherichia coli* L1PEag1

Code	Formulation description	Inhibition zone (mm)
		Escherichia coli L1PEag1
F1	CFS UTNGt28	12.01 ± 0.06
F2	CFS UTNGt2	10.33 ± 0.58
F3	CFS (UTNGt28: UTNGt2) (1:1) (v/v)	11.67 ± 0.58
F4	CFS (UTNGt28: UTNGt2) (1:3) (v/v)	11.33 ± 0.58
F5	CFS (UTNGt28: UTNGt2) (3:1) (v/v)	11.60 ± 0.06
F6	EPS UTNGt28	8.00 ± 0.00
F7	EPS UTNGt2	8.00 ± 0.00
F8	EPS (UTNGt28: UTNGt2) (1:1) (v/v)	8.00 ± 0.00
F9	EPS (UTNGt28: UTNGt2) (1:3) (v/v)	8.00 ± 0.00
F10	EPS (UTNGt28: UTNGt2) (3:1) (v/v)	8.00 ± 0.00
F11	CFS (UTNGt28): EPS UTNGt2) (1:1) (v/v)	9.01 ± 0.06
F12	CFS (UTNGt28): EPS UTNGt2) (1:3) (v/v)	9.01 ± 0.06
FU6	CFS (UTNGt28): EPS UTNGt2) (3:1) (v/v)	12.87 ± 0.06
FU13	CFS UTNGt21O	12.77 ± 0.06

F13	CFS (UTNGt21O: UTNGt28) (1:1) (v/v)	11.33 ± 0.58
F14	CFS (UTNGt21O: UTNGt28) (1:3) (v/v)	11.14 ± 0.06
F15	CFS (UTNGt21O: UTNGt28) (3:1) (v/v)	11.67± 0.58
F16	CFS (UTNGt21O: UTNGt2) (1:1) (v/v)	11.67± 0.58
F17	CFS (UTNGt21O: UTNGt2) (1:3) (v/v)	12.13 ± 0.06
F18	CFS (UTNGt21O: UTNGt2) (3:1) (v/v)	11.67± 0.58
control negative 1	MRS-broth	6.00 ± 0.00
control negative 2	MRSS-broth	6.00 ± 0.00

UTNGt28: strain *Lactococcus lactis* Gt28; UTNGt2: strain *Lactiplantibacillus plantarum* UTNGt2; UTNGt21O: strain *Weissella cibaria* UTNGt21O; CFS: cell free supernatant; EPS: exopolysaccharides.

Table S2

The MIC values against indicator pathogenic bacteria *E. coli* L1PEag1

**Concentration (mg/mL)**	**% of *E. coli* L1PEag1cell reduction**	
	FU6	FU13
1	99.93	98.00
0.5	99.00	97.00
0.25	98.24	26.53
0.125	20.00	18.00

FU6: consists of CFS of UTNGt28 and EPS of UTNGt2 in proportion 3:1 (v/v) (final concentration 100mg/mL); and 2) formulation annotated FU13 and consisting of CFS of UTNGt21O only (final concentration 100 mg/mL). CFS: cell-free supernatant; EPS: exopolysaccharides.
